# Multiple Contrast Tests in the Presence of Partial Heteroskedasticity

**DOI:** 10.1002/bimj.70019

**Published:** 2025-01-13

**Authors:** Mario Hasler, Tim Birr, Ludwig A. Hothorn

**Affiliations:** ^1^ Lehrfach Variationsstatistik Christian‐Albrechts‐University of Kiel Kiel Germany; ^2^ Department of Plant Diseases and Crop Protection Institute of Phytopathology Christian‐Albrechts‐University of Kiel Kiel Germany; ^3^ Retired

**Keywords:** familywise error type I, heteroskedasticity, multiple contrast tests, multivariate distribution, power

## Abstract

This paper proposes a general approach for handling multiple contrast tests for normally distributed data in the presence of partial heteroskedasticity. In contrast to the usual case of complete heteroskedasticity, the treatments belong to subgroups according to their variances. Treatments within these subgroups are homoskedastic, whereas treatments of different subgroups are heteroskedastic. New candidate as well as already existing approaches are described and compared by α‐simulations. Power simulations show that a gain in power is achieved when the partial heteroskedasticity is taken into account compared to procedures which wrongly assume complete heteroskedasticity. The new approaches will be applied to a phytopathological experiment.

## 1 Introduction

Nowadays, multiple contrast tests (MCTs) and corresponding simultaneous confidence intervals are the method of choice for almost all multiple comparison problems. Former multiple comparison procedures may now be considered as special cases of MCTs, or are valid only for special data situations, or they have a worse power or they exceed the familywise error type I (FWE). Although MCTs have a wide application range, we focus here on comparisons of means of normally distributed data. In the case of homoskedasticity, related test statistics jointly follow a multivariate t‐distribution with appropriate degree of freedom and correlation matrix. The use of such a multivariate distribution with dimension equal to the number of contrasts represents the multiplicity adjustment. The incorporation of the correlations of the test statistics under the null hypothesis makes the FWE to be exploited completely; it makes MCTs to be size‐α tests. Hence, the assumption of homoskedasticity is in some ways attributed to mathematical convenience, but it is not always realistic (see Section 2).

Too often, heteroskedasticity is ignored by users because they are not aware of this problem at all, or they are not aware of suitable test procedures. Furthermore, nonstatisticians—as well as many statisticians—try to avoid the application of test procedures for heteroskedastic data. They believe that such procedures would have a bad power compared to test procedures for homoskedastic data. This is an obvious error. The false assumption of homoskedasticity and the use of corresponding test procedures does not necessarily lead to smaller p‐values. Higher p‐values may also appear depending on the data situation, namely, when the groups with smaller variance have smaller sample size (see Section 3 of Hasler and Hothorn 2008, Table 1, Setting (b)). In most situations, the power indeed seems to be improved when homoskedasticity is assumed as corresponding degrees of freedom are anyhow higher than. However, this gain in power is bought with a substantial exceeding of the FWE. Losing the control over the FWE must not be the price to pay for a higher power.

**TABLE 1:** Summary statistics (sample mean, sample variance, group variance, and sample size) for the ZEN values of the mycotoxin data set of Birr et al. (2023).

Spraying technique	Trial location	Sa. mean	Sa. variance	Gr. variance	Sa. size
V1	Ba	497.52	84,635.05	62,031.10	4
V1	He	585.28	49,174.28	62,031.10	4
V1	Ho	478.32	52,283.96	62,031.10	4
V2	Ba	1,965.56	256,303.16	703,488.91	4
V2	He	2,260.34	834,927.58	703,488.91	4
V2	Ho	2,253.57	1,019,235.99	703,488.91	4
V3	Ba	991.48	18,453.55	58,621.17	4
V3	He	648.84	42,016.15	58,621.17	4
V3	Ho	1424.02	115,393.80	58,621.17	4
V4	Ba	657.07	125,209.92	51,056.00	4
V4	He	296.00	8175.27	51,056.00	4
V4	Ho	591.76	19,782.83	51,056.00	4
V5	Ba	556.12	74,933.46	37,191.83	4
V5	He	260.68	13,950.44	37,191.83	4
V5	Ho	585.82	22,691.60	37,191.83	4

In the case of heteroskedasticity, no pooled sample variance is used, but instead the treatment‐specific variances are used for the calculation of the test statistics. Each single test statistic follows approximately a t‐distribution with degree of freedom according to Satterthwaite (1946). The joint distribution is no longer a usual multivariate t‐distribution according to Genz and Bretz (2002) and Hothorn, Bretz, and Genz (2001). This has been an unsolved problem for long time, so approximate approaches had to be taken with conservative or liberal test decisions depending on the amount of heteroskedasticity and the sample allocation (e.g., Games and Howell 1976; Tamhane 1979; Dunnett 1980; Ramsey and Ramsey 2009, …). The PI procedure of Hasler and Hothorn (2008) uses many different multivariate t‐distributions, each with a contrast‐specific degree of freedom according to Satterthwaite (1946) and a correlation matrix based on the sample variances. Hence, each test statistic is compared with “its own” distinct critical value of a multivariate t‐distribution with “its own” degree of freedom to come to a test decision. Thus, the correlations as well as the different marginal distributions of the test statistics are taken into account. That is why the PI procedure is not a max test like the procedure of Herberich, Sikorski, and Hothorn (2010), for example. The latter one is based on the use of HC3 sandwich estimators (Huber 1967; MacKinnon and White 1985). For a critical comparison of the PI procedure and the sandwich procedure of Herberich, Sikorski, and Hothorn (2010), see Hasler (2016). In general, whenever the test statistics follow marginal t‐distributions with different degrees of freedom, these test statistics have different variances. The use of only one multivariate t‐distribution with one degree of freedom leads to one and the same critical value for all the corresponding single test decisions or simultaneous confidence intervals. This causes the simultaneous single tests to have different comparisonwise errors type I. Depending on the relation of sample size and variance per group, liberal or conservative test decisions can be expected then.

Jiang and Ding (2016) developed an extended version of a multivariate t‐distribution: a nonelliptically contoured multivariate t‐distributions, allowing for different marginal degrees of freedom. The authors apply this new distribution in the context of generalized selection‐t, Robit, and linear t mixed‐effects models, respectively. The distribution should also be applicable here in the context of MCTs for heteroskedastic data. However, according to the authors, “the density of the NECTD is very complicated.” The calculation of corresponding distribution parameters is difficult and time‐intensive. Based on the normal mixture representation of their t‐distribution, the authors propose Bayesian inferential procedures based on data augmentation and parameter expansion. For that reason, we still prefer in the following sections the simpler PI procedure according to Hasler and Hothorn (2008). This approach has been proven to be acceptably precise (Hasler and Hothorn 2008; 2018; Hasler 2014); it is faster than the approach of Jiang and Ding (2016), and the usual multivariate t‐distribution, needed for this approach, is already available by package mvtnorm (Genz et al. 2019) of the statistical software R (R Core Team 2023), as well as the PI procedure itself is available by the package SimComp (Hasler and Kluss 2019).

The PI procedure, as well as others too, assume all the treatment groups to have different variances; that is, complete heteroskedasticity is assumed. However, there are often situations where just some treatment groups differ in their variances (see Section 2 for examples). In such cases, there is partial heteroskedasticity and test procedures assuming complete heteroskedasticity must obviously come along with a bad power, whereas test procedures assuming homoskedasticity usually exceed the FWE α. This paper presents and compares adjusted versions of MCTs that explicitly take partial heteroskedasticity into account. In Section 2, two data examples are given. The second data set is taken up again later for illustrations. Some crucial characteristics of Welch's df are considered in Section 3. In Section 4, the testing problem is described, existing test procedures are described and new procedures are introduced. Section 5 presents simulations for the FWE and the power. In Section 6, the example data are evaluated. Section 7 ends with a discussion.

## 2 Examples

**FIGURE 1:**
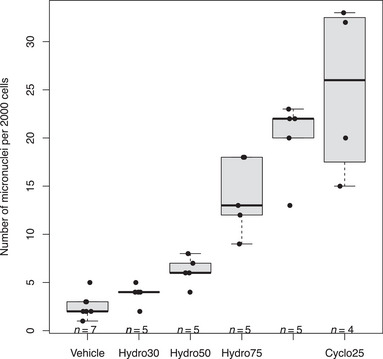
Boxplot of the mutagenicity data set of Adler and Kliesch (1990).

Heteroskedasticity is a widespread problem in data analysis in many scientific areas. Dose‐finding studies, for example, frequently have the problem of heteroskedasticity as the data's variance depends on the dose effects (e.g., see the data in Westfall and Young 1993, 99–101). Data from placebo groups are known to have a higher variance than those of the dose groups (e.g., see the data in Homma, Yamaguchi, and Yamaguchi 2008; Hasler and Hothorn 2012). When data do not strictly follow a normal distribution, heteroskedasticity is present typically. For example, Adler and Kliesch (1990) published data from a micronucleus assay on hydroquinone using a negative control, four doses of hydroquinone and a positive control of 25 mg/kg cyclophosphamide. The goal is to show whether or not the underlying substance is able to induce chromosome damage or interact with the mitotic spindle apparatus. Therefore, the number of micronuclei per animal and 2000 scored cells was measured. Smaller values represent a higher safety of the treatment. This mutagenicity data set is the same as already used in Hauschke et al. (2005), Hasler, Vonk, and Hothorn (2008), and Hasler (2016); and it is available in the R package mratios (Djira et al. 2020). The corresponding variances are clearly not homogeneous. According to Hasler (2012), it is a reasonable assumption that the group variances are bounded by the variances of the two control groups. Hence, heterogeneous group variances are assumed, but in addition nearly equal variances for the noncontrol groups (see Figure 1). The following command in R (R Core Team 2023), package nlme (Pinheiro, Bates, and R Core Team 2020), gives an example for a corresponding model formulation:

library(mratios)

data(Mutagenicity)

Mutagenicity$VG <− as.factor(c(rep(“Vehicle”,7),

rep(“Hydro”,20),rep(“Cyclo”,4)))

library(nlme)

mut.mod <− gls(MN∼Treatment, data = Mutagenicity, weights = varIdent(form=∼1|VG))

**FIGURE 2:**
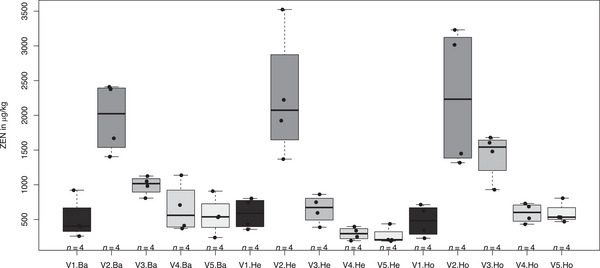
Boxplot of the mycotoxin data set of Birr et al. (2023).

The latter example represents the problem of partial heteroskedasticity in a one‐way ANOVA layout. However, partial heteroskedasticity is even more typical in two‐ or higher‐way ANOVA layouts. In a study by Birr et al. (2023), the efficacy of different fungicide spraying techniques in forage maize was investigated at various trial locations in Northern Germany for the control of mycotoxins produced by the fungal pathogen *Fusarium*. Concentrations (μg/kg) of the mycotoxin zearalenone (ZEN) were measured for the two orthogonal factors trial location (Ba—Barkhorn, He—Hemdingen, Ho—Hohenschulen) and fungicide spraying technique (V1—untreated control, V2—untreated control, V3—overhead spraying technique, V4—dropleg spraying technique, V5—combination of V3 and V4; V2–V5 were artificially inoculated with *Fusarium*). Four observations were made per combination of trial location and spraying technique. Actually, a block factor was within the trial location, but it is ignored here as its effect was just negligibly small. Heteroskedasticity is induced only by spraying technique. In other words, data from the same spraying technique are homoskedastic, data from the same trial location are heteroskedastic as shown in Figure 2. The following command in R (R Core Team 2023), package nlme (Pinheiro, Bates, and R Core Team 2020), gives an example for a corresponding model formulation:

library(nlme)

fert.mod <− gls(ZEN∼ location*technique, data = …, weights = varIdent(form=∼1|technique))

Table 1 shows the summary statistics. Both the naive sample variances (Sa. variance) are shown, implying a complete heteroskedasticity, as well as the model‐based group variances (Gr. variance), implying partial heteroskedasticity.

## 3 Some Characteristics of Welch's df

The degree of freedom $df^{Satt}$ according to Satterthwaite (1946) represents the extension of the degree of freedom $df^{Welch}$ according to Welch (1938) to the case of any linear combination of more than two mean estimates. The other way round, $df^{Welch}$ can be seen as a special case of $df^{Satt}$ for only two, equally weighted mean estimates

(1) $$df^{Welch}=\frac{{\left(S_{1}^{2}/n_{1}+S_{2}^{2}/n_{2}\right)}^{2}}{\frac{{\left(S_{1}^{2}/n_{1}\right)}^{2}}{n_{1}-1}+\frac{{\left(S_{2}^{2}/n_{2}\right)}^{2}}{n_{2}-1}}=\frac{{\left(\frac{1}{n_{1}}+\frac{S_{2}^{2}}{S_{1}^{2}}\frac{1}{n_{2}}\right)}^{2}}{\frac{{\left(\frac{1}{n_{1}}\right)}^{2}}{n_{1}-1}+\frac{{\left(\frac{S_{2}^{2}}{S_{1}^{2}}\frac{1}{n_{2}}\right)}^{2}}{n_{2}-1}},$$

with the sample sizes $n_{1}$ and $n_{2}$ and the sample variances $S_{1}^{2}$ and $S_{2}^{2}$ of two corresponding treatments. We consider $df^{Welch}$ here for reasons of simplicity. Its characteristics can be carried over to $df^{Satt}$.

**FIGURE 3:**
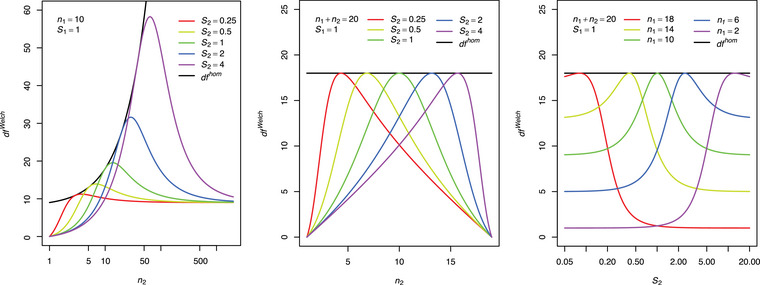
Dependency of $df^{Welch}$ on sample sizes and standard deviations.

Indeed, there are some properties of $df^{Welch}$ which are strange at first glance. Of course, $df^{Welch}$ depends on samples sizes. In contrast to the related degree of freedom $df^{hom}=n_{1}+n_{2}-2$ of a usual two‐sample t‐test, $df^{Welch}$ also depends on variance estimates. But actually, their ratio is important, the variance estimates themselves and their difference are not. See the right expression of Equation (1). The left part of Figure 3 shows the dependency of $df^{Welch}$ on the sample size $n_{2}$ and on the ratio of the standard deviations $S_{1}$ and $S_{2}$. To the right (9) and to the left (0) of the curve maximum, $df^{Welch}$ converges to the degree of freedom of a one‐sample t‐test of the group with the smaller sample size, irrespective of the standard deviation of this group

$$\min\left\{n_{1},n_{2}\right\}-1\leq df^{Welch}\leq df^{hom}.$$

An unexpected practical consequence is that $df^{Welch}$ may substantially decrease although the total sample size $N=n_{1}+n_{2}$ is increasing. Moreover, a commonly accepted conviction of many statisticians is that $df^{Welch}$ coincides with $df^{hom}$ if the two variance estimates are equal. However, this is not correct in a general sense. Only in a homoskedastic ($S_{1}=S_{2}$), balanced ($n_{1}=n_{2}$) situation (green), $df^{Welch}$ equals the usual $df^{hom}$. In other words, even if the standard deviations of two groups are exactly equal, $df^{Welch}<df^{hom}$, except for the case of equal sample sizes, where $df^{Welch}=df^{hom}$. Therefore, the question arises, whether $df^{Welch}$ can also coincide with $df^{hom}$ for unbalanced sample sizes in heteroskedastic situations. Indeed, it can as the colored lines ($df^{Welch}$) are tangent to the black line ($df^{hom}$). More obviously, this fact can be seen in the middle part of Figure 3. In contrast to the figure's left part, the total sample size is fixed here: $N=n_{1}+n_{2}=20$. The maximum degree of freedom $df^{Welch}=df^{hom}=18$ is achieved depending on the variance ratio. Only in a homoskedastic ($S_{1}=S_{2}$) situation, the maximum degree of freedom $df^{Welch}=df^{hom}$ is achieved for balanced ($n_{1}=n_{2}$) sample sizes. In other words, for specific heteroskedastic situations, the maximum degree of freedom $df^{Welch}=df^{hom}$ can also be achieved, however, only for unbalanced sample sizes. The group with the higher standard deviation must have the higher sample size. Finally, the right part of Figure 3 shows the dependency of $df^{Welch}$ on the standard deviation $S_{2}$ and several sample size allocations for a fixed sample size. The degree of freedom $df^{Welch}$ converges to the degree of freedom of a one‐sample t‐test of the group with the higher standard deviation. The yellow curve, for example, converges to value $n_{2}-1=13$ (left side of the curve maximum) and $n_{1}-1=5$ (right side of the maximum).

In sum: the higher the samples size or the smaller the standard deviation of one group, the more this group seems to play the role of a scalar quantity. Only the other group (with the smaller sample size or the higher standard deviation) fulfills the role of a random variable. Hence, a corresponding Welch t‐test “converges” to a one‐sample t‐test.

## 4 Testing Problem and Test Procedures

### 4.1 Testing Problem

We assume a one‐way layout with a certain number of treatments. Each treatment belongs to one certain variance group. The variance groups can consist of different numbers of treatments. For h=1,⋯,H, $i=1,\cdots ,I_{h}$, and $j=1,\cdots ,n_{hi}$, let $X_{hij}$ denote the jth observation under the ith treatment of the hth variance group in a one‐way layout. Hence, there are $\sum_{h=1}^{H}I_{h}$ treatments altogether. Suppose the $X_{hij}$ to be independently normal with means $\mu_{hi}$ and variances $\sigma_{h}^{2}$, thus

$$X_{hij}∼⊥N\left(\mu_{hi},\sigma_{h}^{2}\right)\;\left(h=1,\cdots ,H,\;i=1,\cdots ,I_{h},\;j=1,\cdots ,n_{hi}\right).$$

For the case that each variance group contains only one treatment ($I_{h}=1\;\forall h=1,\cdots ,H$), there is complete heteroskedasticity and index i is redundant. Otherwise, there is partially heteroskedasticity. A corresponding model formulation is given by

$$\begin{array}{ccc} X_{hij} & = & \bar{\mu }+\alpha_{hi}+\varepsilon_{hij},\;\varepsilon_{hij}∼⊥N\left(0,\sigma_{h}^{2}\right)\\ & & \;(h=1,\cdots ,H,\;i=1,\cdots ,I_{h},\;j=1,\cdots ,n_{hi}), \end{array}$$

where $\bar{\mu }$ is the overall expected value for all observations, $\alpha_{hi}$ is the effect of treatment hi, and $\mu_{hi}=\bar{\mu }+\alpha_{hi}$. The residuals $\varepsilon_{hij}$ are independent and follow a normal distribution with expected value zero and variances $\sigma_{h}^{2}$. Let $\mu ={\left(\mu_{11},\cdots ,\mu_{HI_{H}}\right)}^{\prime }$ be the vector of treatment means and $\bar{X}={\left({\bar{X}}_{11},\cdots ,{\bar{X}}_{HI_{H}}\right)}^{\prime }$ its estimator with the usual unbiased mean estimators ${\bar{X}}_{hi}$. The sample variances are given by

(2) $$S_{h}^{2}=\frac{\sum_{i=1}^{I_{h}}\sum_{j=1}^{n_{hi}}{\left(X_{hij}-{\bar{X}}_{hi}\right)}^{2}}{\sum_{i=1}^{I_{h}}\left(n_{hi}-1\right)}\;h=1,\cdots ,H.$$

We are interested in the vector of contrasts $\eta ={\left(\eta_{1},\cdots ,\eta_{L}\right)}^{\prime }$, where

$$\eta_{l}=\sum_{h=1}^{H}\sum_{i=1}^{I_{h}}c_{lhi}\mu_{hi}\;\left(l=1,\cdots ,L\right).$$

The vectors $c_{l}={\left(c_{l11},\cdots ,c_{lHI_{H}}\right)}^{\prime }$ consist of real constants with $\sum_{h=1}^{H}\sum_{i=1}^{I_{h}}c_{lhi}=0\;\left(l=1,\cdots ,L\right)$. The objective is to test the L hypotheses

(3) $$H_{0l}:\eta_{l}\leq \delta_{l}\;\left(l=1,\cdots ,L\right)$$

for specified absolute thresholds $\delta_{l}$. Usually, $\delta_{l}=0$ for all l=1,⋯,L. This testing problem is a union‐intersection test because the overall null hypothesis of interest can be expressed as an intersection of these local null hypotheses, that is,

$$H_{0}=\cap_{l=1}^{L}H_{0l}.$$

Corresponding test statistics $T={\left(T_{1},\cdots ,T_{L}\right)}^{\prime }$ are given by

$$T_{l}=\frac{{\hat{\eta }}_{l}-\delta_{l}}{\sqrt{\sum_{h=1}^{H}\sum_{i=1}^{I_{h}}c_{lhi}^{2}S_{h}^{2}/n_{hi}}}\;\left(l=1,\cdots ,L\right).$$

The corresponding degrees of freedom according to Satterthwaite (1946) are given by

$$df_{l}^{Satt}=\frac{{\left(\sum_{h=1}^{H}\sum_{i=1}^{I_{h}}\frac{c_{lhi}^{2}S_{hi}^{2}}{n_{hi}}\right)}^{2}}{\sum_{h=1}^{H}\sum_{i=1}^{I_{h}}\frac{c_{lhi}^{4}S_{hi}^{4}}{n_{hi}^{2}\left(n_{hi}-1\right)}}\;\left(l=1,\cdots ,L\right).$$

These degrees of freedom are appropriate for the case of complete heteroskedasticity, they refer to the variance estimates $S_{hi}^{2}$, instead of $S_{h}^{2}$. However, in the case of partial heteroskedasticity, we introduce

(4) $$df_{l}=\frac{{\left(\sum_{h=1}^{H}\frac{{\left(\sum_{i=1}^{I_{h}}\left|c_{lhi}\right|\right)}^{2}S_{h}^{2}}{N_{h}}\right)}^{2}}{\sum_{h=1}^{H}\frac{{\left(\sum_{i=1}^{I_{h}}\left|c_{lhi}\right|\right)}^{4}S_{h}^{4}}{N_{h}^{2}\left(N_{h}-I_{h}\right)}}\;\left(l=1,\cdots ,L\right),$$

where $N_{h}=\sum_{i=1}^{I_{h}}n_{hi}$. Equation (4) is a natural extension of the degree of freedom according to Satterthwaite (1946) to the case of many treatments sharing the same variance group. If each variance group would contain only one treatment ($I_{h}=1\;\forall h=1,\cdots ,H$), index i is redundant and $df_{l}$ coincides with the usual $df_{l}^{Satt}$.

The estimated correlation matrix of the test statistics $T_{1},\cdots ,T_{L}$ under $H_{0}$ is given by the elements

(5) $$\rho_{ll^{\prime }}=\frac{\sum_{h=1}^{H}\sum_{i=1}^{I_{h}}c_{lhi}c_{l^{\prime }hi}S_{h}^{2}/n_{hi}}{\sqrt{\sum_{h=1}^{H}\sum_{i=1}^{I_{h}}c_{lhi}^{2}S_{h}^{2}/n_{hi}}\sqrt{\sum_{h=1}^{H}\sum_{i=1}^{I_{h}}c_{l^{\prime }hi}^{2}S_{h}^{2}/n_{hi}}}\;\left(1\leq l,l^{\prime }\leq L\right).$$

Hence, under $H_{0}$, $T_{l}$ approximately follows a t‐distribution with $df_{l}$ degrees of freedom and $T_{1},\cdots ,T_{L}$ have a correlation matrix $R={\left(\rho_{ll^{\prime }}\right)}_{l,l^{\prime }}$.

### 4.2 Existing and New Test Procedures

We refer to the mycotoxin data set of Birr et al. (2023) from Section 2 as an illustrating example here. A corresponding model (see Section 2) results in 15 mean estimates and five variance estimates. Measurement values of the treatments V1Ba, V1He, and V1Ho have the same theoretical variance $\sigma_{V1}^{2}$, …, measurement values of the treatments BaV5Ba, V5He, and V5Ho have the same theoretical variance $\sigma_{V5}^{2}$, where $\sigma_{V1}^{2}\neq \cdots \neq \sigma_{V5}^{2}$. The five spraying techniques are of interest to be compared, split for the three trial locations.

A wide range of more or less good evaluation strategies exists for such a data situation. Especially in R, one would probably use the command glht(), provided by the package multcomp (Hothorn et al. 2021), to conduct an appropriate MCT. For such a gls() model (see Section 2), the command glht() applies a multivariate normal distribution by default as no unique single degree of freedom can be obtained from the model to carry over for a potential multivariate t‐distribution. However, glht() allows the manual setting of a single degree of freedom. Typically, one is willing to use the single residual degree of freedom, used by the anova() command too, which is 45 here. This procedure is denoted as **SDF** here. Unfortunately, the command gls() (package nlme Pinheiro, Bates, and R Core Team 2020) is not known by some users. They just run the usual command lm() for linear models in R, requiring homoskedasticity. The command glht() provides an option vcov where the HC3 sandwich estimator Herberich, Sikorski, and Hothorn (2010) can be chosen for calculations of corresponding MCTs to make them robust against heteroskedasticity. This procedure is denoted as **SW** here. For a critical view on the use of sandwich estimators for MCTs, see Hasler (2016). Both approaches are not completely sufficient to adjust MCTs for complete or partial heteroskedasticity, although they are widely used. The degree of freedom is too high. Moreover, the test decisions are based on the same critical value for all test statistics. This causes different comparisonwise errors type I. Depending on the relation of sample size and variance per group, liberal or conservative test decisions can be expected naturally (Hasler 2016).

**FIGURE 4:**
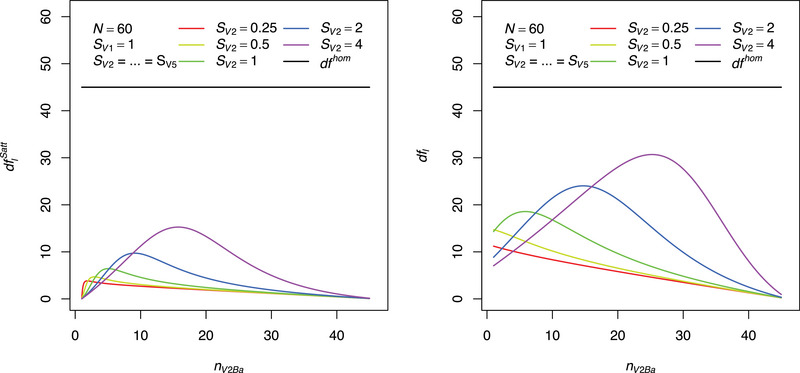
Degrees of freedom for the first comparison V2Ba–V1Ba of the mycotoxin data set of Birr et al. (2023) based on **PIa** (left) and **PH** (right) for several values of the standard deviation $S_{V2}$, depending on the sample size $n_{V2Ba}$.

A further idea is to do an MCT based on the PI procedure according to Hasler and Hothorn (2008) assuming a complete heteroskedasticity. The variance estimates $S_{hi}^{2}$, instead of $S_{h}^{2}$, are used which is clearly not optimal. With the knowledge of equal variances of treatments belonging to the same spraying technique, the variance estimates (2) are best linear unbiased estimators. Hence, a simple better, promising idea is still to use **PI** but with the adaption that the estimates $S_{h}^{2}$ are used instead of $S_{hi}^{2}$ for the calculations of corresponding test statistics, their correlations under $H_{0}$ and degrees of freedom. This procedure is referred here to as **PIa**. This procedure should lead to an improved behavior compared to **PI** as it clearly reflects the situation of partial heteroskedasticity better. The left part of Figure 4 shows the degrees of freedom for the first comparison V2Ba–V1Ba based on **PIa** depending on the sample size $n_{V2Ba}$ of treatment V2Ba and the sample standard deviation $S_{V2}$. The remaining treatments have balanced sample sizes. One can see that there is a big gap between these degrees of freedom and the usual one of MCTs for homoskedastic data ($df^{hom}$). Even if the sample variances are totally equal (green), the degrees of freedom based on **PIa** are much smaller.

In contrast to the procedures **PI** or **PIa**, procedure **PH** is defined such that multivariate t‐distributions with degrees of freedom (4) and a correlation matrix with elements (5) are used for the calculation of critical values or p‐values. The degrees of freedom (4) should also be appropriate for the approach of Jiang and Ding (2016) which is not considered here for reasons given in Section 1. In the same way as the left part of Figure 4, the right part shows the degrees of freedom of the new procedure **PH** based on Equation (4). One can see that **PH** results in much higher degrees of freedom, nearer to those of usual MCTs for homoskedastic data ($df^{hom}$), as compared to **PIa**.

Depending on the amount of heteroskedasticity and the sample allocation, there are situations where a simple variance adaption (**PIa**) leads to higher degrees of freedom than for **PH** (see Section 3). Therefore, define **PHmax** as an extension of **PH**, where the maximum of the degrees of freedom corresponding to **PIa** and **PH** is used.

### 4.3 Simultaneous Confidence Intervals

For all the procedures considered in Section 4.2, corresponding approximate (1−α)100% simultaneous confidence intervals are available for $\eta_{1},\cdots ,\eta_{L}$. This is in fact a crucial advantage of MCTs over other multiple comparison procedures. The lower bounds for procedure **PH** are obtained as

$${\hat{\eta }}_{l}^{lower}=\left(\sum_{h=1}^{H}\sum_{i=1}^{I_{h}}c_{lhi}{\bar{X}}_{hi}\right)-t_{L,df_{l},R,1-\alpha }\sqrt{\sum_{h=1}^{H}\sum_{i=1}^{I_{h}}c_{lhi}^{2}S_{h}^{2}/n_{hi}}\;\left(1\leq l\leq L\right)$$

with the lower (1−α) quantiles $t_{L,df_{l},R,1-\alpha }$ of the L‐variate t‐distributions. Note that the lower bounds are somewhat different for the other procedures considered in Section 4.2 as different sample variances and different (1−α) quantiles are used. These lower bounds can be used for the statistical problem (3). $H_{0l}$ is rejected for a given level α if ${\hat{\eta }}_{l}^{lower}>\delta_{l}$.

## 5 α‐ and Power Simulations

All the methods described in Section 4 are approximate ones. Simulations concerning the FWE and the power are needed for a validation of their quality. The ususal MCT for homoskedastic data (**HOM**) was additionally considered for these simulations. In order to cover situations representing one‐way and multiway layouts, the following settings were chosen. Six treatments were taken to compare. The first treatment was regarded as the negative control. Five MCT problems which are all related to hypotheses (3) were considered: Dunnett (comparisons vs. a control), Tukey (all‐pair comparisons), Williams (trend tests), Average (comparisons vs. the average of the remaining groups) and a user‐defined contrast test with contrast matrix

$$C=\left(\begin{array}{cccccc} -1, & 0, & 1, & 0, & 0, & 0\\ 0, & -1, & 0, & 1, & 0, & 0\\ -1, & 0, & 0, & 0, & 1, & 0\\ 0, & -1, & 0, & 0, & 0, & 1\\ 0, & 0, & -1, & 0, & 1, & 0\\ 0, & 0, & 0, & -1, & 0, & 1\\ -1, & 1, & 0, & 0, & 0, & 0\\ 0, & 0, & -1, & 1, & 0, & 0\\ 0, & 0, & 0, & 0, & -1, & 1 \end{array}\right).$$

The latter contrast test refers to a two‐way layout where the first six contrasts represent comparisons for the three levels of a first influence factor, the three last contrast represent comparisons for the two levels of a second influence factor; all comparisons are split for the levels of the remaining factor. All these MCT problems were considered as one‐sided problems here for reasons of consistency although some are rather two‐sided. The nominal level of the FWE was 0.05. Four different settings were considered; each had a total sample size of 60. They are:

(a) a balanced allocation; the last groups have the highest standard deviation:
$n_{1}=\cdots =n_{6}=10$ and $\sigma_{1}=\sigma_{2}=10,\sigma_{3}=\sigma_{4}=30,\sigma_{5}=\sigma_{6}=50$
(b) the first group (control) has the smallest sample size; the last groups have the highest standard deviation:
$n_{1}=5,n_{2}=\cdots =n_{6}=11$ and $\sigma_{1}=\sigma_{2}=10,\sigma_{3}=\sigma_{4}=30,\sigma_{5}=\sigma_{6}=50$
(c) the last group has the smallest sample size; the last groups have the highest standard deviation:
$n_{1}=\cdots =n_{5}=11,n_{6}=5$ and $\sigma_{1}=\sigma_{2}=10,\sigma_{3}=\sigma_{4}=30,\sigma_{5}=\sigma_{6}=50$
(d) a balanced allocation; the homoskedastic case:
$n_{1}=\cdots =n_{6}=10$ and $\sigma_{1}=\cdots =\sigma_{6}=30$.

The expectation value for all groups was 100. All simulation results were obtained by 100,000 simulation runs, using a program code in the statistical software R (R Core Team 2023).

**FIGURE 5:**
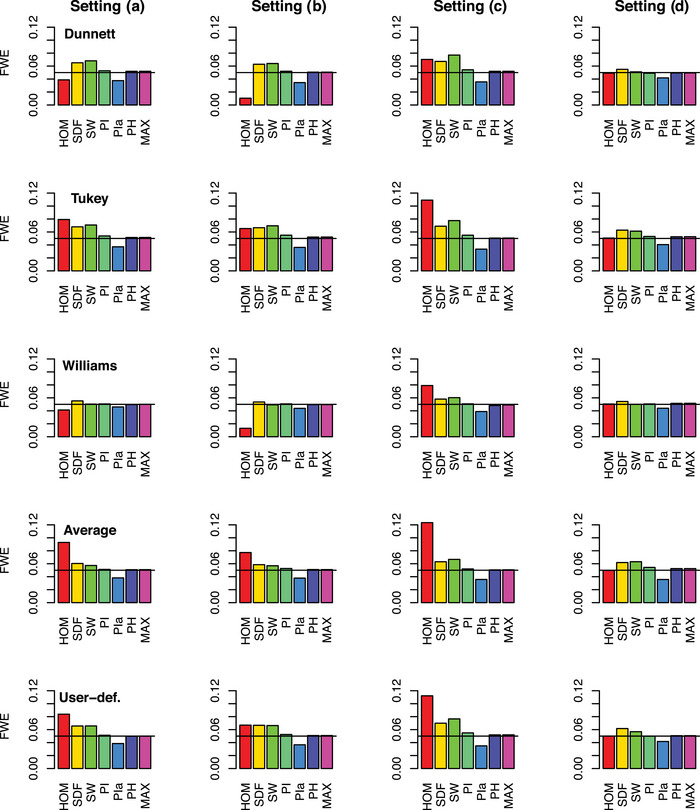
Simulated global FWE of MCT for I=6 treatments, several contrasts, procedures, and settings; α=0.05.

Figure 5 shows the results of the simulations concerning the FWE. As expected, **HOM** tends to either conservatism or liberalism, respectively, depending on the setting and the contrast (from 0.0105, (b), Dunnett to 0.1234, (c), Average). In general, setting (c)—combining the smallest sample size and the highest standard deviation—leads to especially liberal behavior. On the other hand, **HOM** is very conservative for setting (b), Dunnett and Williams contrasts. The comparison of settings (a) and (b) for **HOM** shows that a balanced allocation generally increases the FWE when heteroskedasticity is present but ignored. This can lead to less conservative (0.0388 instead of 0.0105) or more liberal (0.0929 instead of 0.0773) behavior. **SDF** exceeds the nominal α‐level, slightly or clearly, for all settings and contrasts (from 0.0537, (b), Williams to 0.0699, (c), User‐def.). **SW** exceeds the nominal α‐level for almost all settings and contrasts (from 0.0492, (b), Williams to 0.0776, (c), Tukey). Its range is wider than for **SDF**. Furthermore, note that all procedures where the test decisions are based on only one, the same critical value for all test statistics (**HOM**, **SDF**, **SW**) additionally cause different comparisonwise errors type I. This obvious fact cannot be seen within Figure 5 as only the global FWE was simulated. Therefore, these procedures cannot be recommended. **PI** slightly exceeds the α‐level for some settings and contrasts (from 0.0490, (d), Dunnett to 0.0550, (b), Tukey). **PIa** maintains the α‐level but it falls below it for all settings and contrasts (0.0335, (c), Tukey ‐ 0.0460, (a), Williams). **PIa** is also constantly below **SDF** as both procedures mainly differ in the degrees of freedom, which are always smaller for **PIa**. With few exceptions, **PH** maintains the α‐level exactly (from 0.0482, (c), Willimas to 0.0526, (d), Tukey). Hence, the use of the correct variance estimates $S_{h}^{2}$ instead of $S_{hi}^{2}$—the theoretical advantage of **SDF** and **PIa** over **PI**—is not sufficient without a corresponding adjustment of the degrees of freedom. **PHmax** does not significantly differ from **PH** (from 0.0490, (c), Willimas to 0.0527, (d), Tukey).

Because of heteroskedasticity, adjustments of the degrees of freedom and of the correlations between the test statistics are necessary. This means that the critical values of **PH** for test decisions or simultaneous confidence intervals are, in fact, random variables because they depend on the sample values. On the other hand, the test statistic $T_{l}\left(l=1,\cdots ,L\right)$ will not be compared with the same critical value. Different multivariate t‐distributions with different degrees of freedom are applied. Power calculations are therefore not possible so far. Power comparisons by simulations are possible, however. The different α‐levels of the procedures considered above do not permit a fair power comparison, especially for situations where the procedures do not maintain the FWE. Nevertheless, power is an important dimension. A simulation study about the complete (all‐pairs) power has been performed with a similar setting as for the FWE, where only Dunnett and Tukey contrasts were considered. The mean of the third and the fifth treatment were similarly increased so that the Dunnett contrasts $\eta_{2}=\eta_{4}$ and the Tukey contrasts $\eta_{2}=\eta_{4}=\eta_{6}=\eta_{8}=\eta_{13}$ varied from 30 to 70. (Again, note that these MCT problems were considered as one‐sided problems here for reasons of consistency.) All simulation results were obtained by 100,000 simulation runs.

**FIGURE 6:**
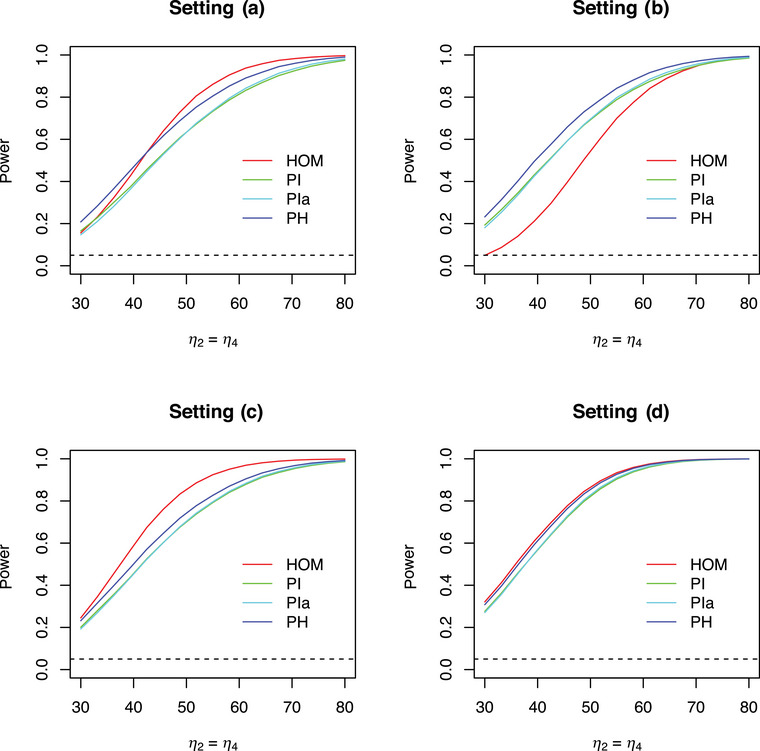
Simulated complete (all‐pairs) power of MCT for I=6 treatments, Dunnett contrasts, several procedures and settings; α=0.05.

**FIGURE 7:**
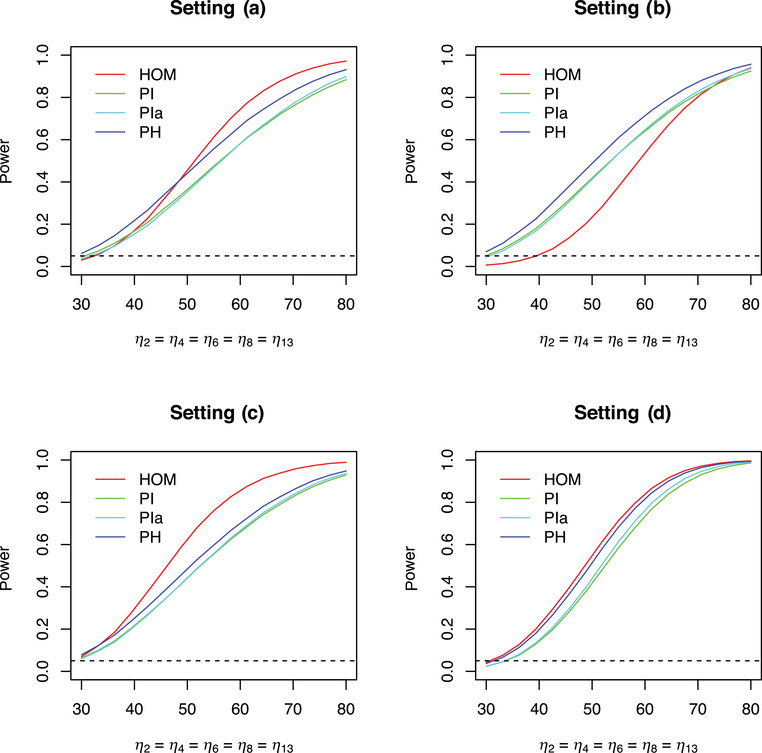
Simulated complete (all‐pairs) power of MCT for I=6 treatments, Tukey contrasts, several procedures and settings; α=0.05.

Figures 6 and 7 show the corresponding results. As there were no differences between **PH** and **PHmax** for these power simulations, the latter one is not displayed here. Comparing the two figures, one can see that the behavior of the procedures considered is approximately the same with regard to the specific contrasts. **PH** always achieves a better power compared to **PI** and **PIa** for all settings. The maximum gain in power of **PH** over **PI** for these simulations is 0.09969 (setting (d), Tukey contrast). **PI** and **PIa** are always very close to each other. Again, the use of the correct variance estimates $S_{h}^{2}$ instead of $S_{hi}^{2}$ is hence not sufficient without a corresponding adjustment of the degrees of freedom. The procedures **HOM**, **SDF**, **SW** do not maintain the α‐level in general. Hence, a comparison of their power behavior with that of **PH** is not fair. However, it is interesting to see that **HOM** achieves a very bad power for setting (b). This is because the first group (control) has the smallest standard deviation here. **HOM**, however, assigns a higher standard deviation to this group. In a Dunnett (Tukey) contrast, this group appears in all (many) contrasts, leading to conservative test decisions. Hence, the naive idea that ignoring heteroskedasticity would generally lead to a better power is not correct.

## 6 Evaluation of the Example

We refer to the example in Section 2. A suitable model is estimated, resulting in 15 mean estimates. Such a complete model can always be transformed to a pseudo one‐way (or cell means) model (Schaarschmidt and Vaas 2009). An artificial pseudo factor covers all combinations of the actual factors, resulting in the levels V1Ba, V1He, Va1Ho, …V5Ba, V5He, Va5Ho. To simplify matters, there is interest in a comparison of the four spraying techniques V2,…,V5 versus the control technique V1, split for the trial locations, that is, 12 comparisons: V2Ba versus V1Ba, V2He versus V1He, V2Ho versus V1Ho …V5Ba versus V1Ba, V5He versus V1He, V5Ho versus V1Ho. An MCT is needed with the contrast matrix

$$\begin{array}{cc} C=\left(\begin{array}{ccccccccccccccc} -1, & 0, & 0, & 1, & 0, & 0, & 0, & 0, & 0, & 0, & 0, & 0, & 0, & 0, & 0\\ 0, & -1, & 0, & 0, & 1, & 0, & 0, & 0, & 0, & 0, & 0, & 0, & 0, & 0, & 0\\ 0, & 0, & -1, & 0, & 0, & 1, & 0, & 0, & 0, & 0, & 0, & 0, & 0, & 0, & 0\\ -1, & 0, & 0, & 0, & 0, & 0, & 1, & 0, & 0, & 0, & 0, & 0, & 0, & 0, & 0\\ 0, & -1, & 0, & 0, & 0, & 0, & 0, & 1, & 0, & 0, & 0, & 0, & 0, & 0, & 0\\ 0, & 0, & -1, & 0, & 0, & 0, & 0, & 0, & 1, & 0, & 0, & 0, & 0, & 0, & 0\\ -1, & 0, & 0, & 0, & 0, & 0, & 0, & 0, & 0, & 1, & 0, & 0, & 0, & 0, & 0\\ 0, & -1, & 0, & 0, & 0, & 0, & 0, & 0, & 0, & 0, & 1, & 0, & 0, & 0, & 0\\ 0, & 0, & -1, & 0, & 0, & 0, & 0, & 0, & 0, & 0, & 0, & 1, & 0, & 0, & 0\\ -1, & 0, & 0, & 0, & 0, & 0, & 0, & 0, & 0, & 0, & 0, & 0, & 1, & 0, & 0\\ 0, & -1, & 0, & 0, & 0, & 0, & 0, & 0, & 0, & 0, & 0, & 0, & 0, & 1, & 0\\ 0, & 0, & -1, & 0, & 0, & 0, & 0, & 0, & 0, & 0, & 0, & 0, & 0, & 0, & 1 \end{array}\right) & \begin{array}{c} \;\;\;\;\to \eta_{1}=\mu_{V2Ba}-\mu_{V1Ba}\\ \;\;\;\;\to \eta_{2}=\mu_{V2He}-\mu_{V1He}\\ \;\;\;\;\to \eta_{3}=\mu_{V2Ho}-\mu_{V1Ho}\\ \;\;\;\;\cdots \\ \;\;\;\;\cdots \\ \;\;\;\;\cdots \\ \;\;\;\;\cdots \\ \;\;\;\;\cdots \\ \;\;\;\;\cdots \\ \;\;\;\;\cdots \\ \;\;\;\;\cdots \\ \;\;\;\;\to \eta_{12}=\mu_{V5Ho}-\mu_{V1Ho} \end{array} \end{array}$$

Table 2 shows the results (adjusted p‐values and degrees of freedom) of the MCT for the ZEN values of the mycotoxin data set of Birr et al. (2023) applying the several procedures. One can see that comparisons with the same spraying techniques but different trial locations (first three comparisons, second three comparisons, ⋯ in Table 2) get the same degree of freedom by the procedures **PIa**, **PH**, and **PHmax**. This is nothing but fair, because the three trial locations do not cause different variances here. Depending on the heteroskedasticity assumption, different results can be observed. The comparison V3–V1:Ho is found to be significant by all procedures. The first three comparisons are abundantly clear (see the corresponding mean differences). Except for **PI** and **PIa**, all these comparisons are significant. The very bad power of **PIa** here is very surprising. It is even worse than for **PI**. The tendential difference of comparison V3 ‐ V1:Ba is wrongly declared to be significant by **SDF**.

**TABLE 2:** p‐values of the tests for the ZEN values of the mycotoxin data set of Birr et al. (2023) applying the several procedures; values in parentheses are the corresponding degrees of freedom.

Comparison	HOM	SDF	SW	PI	PIa	PH/PHmax
V2–V1:Ba	**0.0001**	**0.0089**	**0.0005**	**0.0260**	0.1333	**0.0340**
1468.04	(45)	(45)	(45)	(4.79)	(3.52)	(10.57)
V2–V1:He	< **0.0001**	**0.0023**	**0.0181**	0.1127	0.0971	**0.0160**
1675.06	(45)	(45)	(45)	(3.35)	(3.52)	(10.57)
V2–V1:Ho	< **0.0001**	**0.0011**	**0.0245**	0.1232	0.0840	**0.0112**
1775.25	(45)	(45)	(45)	(3.31)	(3.52)	(10.57)
V3–V1:Ba	0.3885	**0.0351**	0.0527	0.1218	0.1304	0.0543
493.96	(45)	(45)	(45)	(4.25)	(6.00)	(17.99)
V3–V1:He	0.9790	0.9723	0.9680	0.9590	0.9718	0.9722
63.56	(45)	(45)	(45)	(5.96)	(6.00)	(17.99)
V3–V1:Ho	**0.0162**	< **0.0001**	**0.0013**	**0.0221**	**0.0116**	**0.0002**
945.70	(45)	(45)	(45)	(5.26)	(6.00)	(17.99)
V4–V1:Ba	0.9301	0.8067	0.9219	0.8913	0.8040	0.8058
159.55	(45)	(45)	(45)	(5.78)	(5.94)	(17.83)
V4–V1:He	1.0000	1.0000	1.0000	1.0000	1.0000	1.0000
−289.28	(45)	(45)	(45)	(3.97)	(5.94)	(17.83)
V4–V1:Ho	0.9593	0.9112	0.8829	0.8360	0.9074	0.9103
113.44	(45)	(45)	(45)	(4.99)	(5.94)	(17.83)
V5–V1:Ba	0.9804	0.9716	0.9801	0.9763	0.9711	0.9714
58.60	(45)	(45)	(45)	(5.98)	(5.65)	(16.94)
V5–V1:He	1.0000	1.0000	1.0000	1.0000	1.0000	1.0000
−324.60	(45)	(45)	(45)	(4.58)	(5.65)	(16.94)
V5–V1:Ho	0.9622	0.9089	0.9000	0.8600	0.9051	0.9079
107.50	(45)	(45)	(45)	(5.19)	(5.65)	(16.94)

p‐values smaller 0.05 usually represent significant/important effects in statistics.

## 7 Discussion

It is hard to decide in practice if data are homo‐, hetero‐ or partially heteroskedastic. Such a decision should, in the best case, be made before the test results are observed in order to avoid a situation in which the statistical analysis becomes data‐driven. If this is not possible, we recommend to draw this decision upon an appropriate statistical model with a corresponding residual analysis (Kozak and Piepho 2018). However, misspecification can still occur. The specific behavior of the new method **PH** depending on the type or amount of misspecification is also probably worth a further investigation. If the variance situation is misspecified by the user, the results of the new method **PH** concerning the FWE and power should be bounded by the methods **HOM** and **PI**. The robustness of the new method **PH** definitely depends on the ratio of homoskedasticity and heteroskedasticity within the data, and it depends on the particular misspecification of the user. The mildest case of misspecification is when the data are in fact homoskedastic. The FWE is then maintained, but the power is smaller. A scenario where the data are in fact completely heteroskedastic represents a middle case of misspecification. Only a certain part of the heteroskedasticity is then correctly taken into account. A kind of worst case may obviously occur if a prespecification claims a certain factor (e.g., trial location) to cause heteroskedasticity, whereas a different factor (e.g., spraying technique) is in fact the cause. Then, the actually right method works in the absolutely wrong direction. In a further simulation study (we do not present details here), the method **PH** had an increased FWE (0.11574, Average contrast) in this latter situation.

The **PH** procedure described in this article still has the assumptions that the data are normally distributed and come from a completely randomized design. If data are not normally distributed, if they come from more complex experimental designs (block design, split plot design, …), or if covariates are included, the **PH** procedure cannot be used. However, the general approach of adjusted degrees of freedom can also be adopted in generalized linear models (McCullagh and Nelder 1989), mixed models (Pinheiro and Bates 2000), or ANCOVA models (Cochran 1957), respectively. An application to the latter models could be a next, interesting step. For example, current multiple comparisons based on mixed models are usually based on the **SDF** method leading to liberal or conservative test decisions depending on the degree‐of‐freedom approximation (see Faes et al. 2009, for example). Furthermore, nonparametric MCTs and corresponding simultaneous confidence intervals are also available for nonnormally distributed data according to (Konietschke, Hothorn, and Brunner 2012). This procedure is also robust for heteroskedasticity.

## Conflicts of Interest

The authors declare no conflicts of interest.

## Supporting information


Supporting Information

## Data Availability

The data that support the findings of this study are available in the supplementary material of this article.
