# Supplementary figures and images for: Multiple Contrast Tests in the Presence of Partial Heteroskedasticity
Source: Biom J. 2025 Jan 13;67(1):e70019. doi: 10.1002/bimj.70019 (PMC11729621; doi:10.1002/bimj.70019)

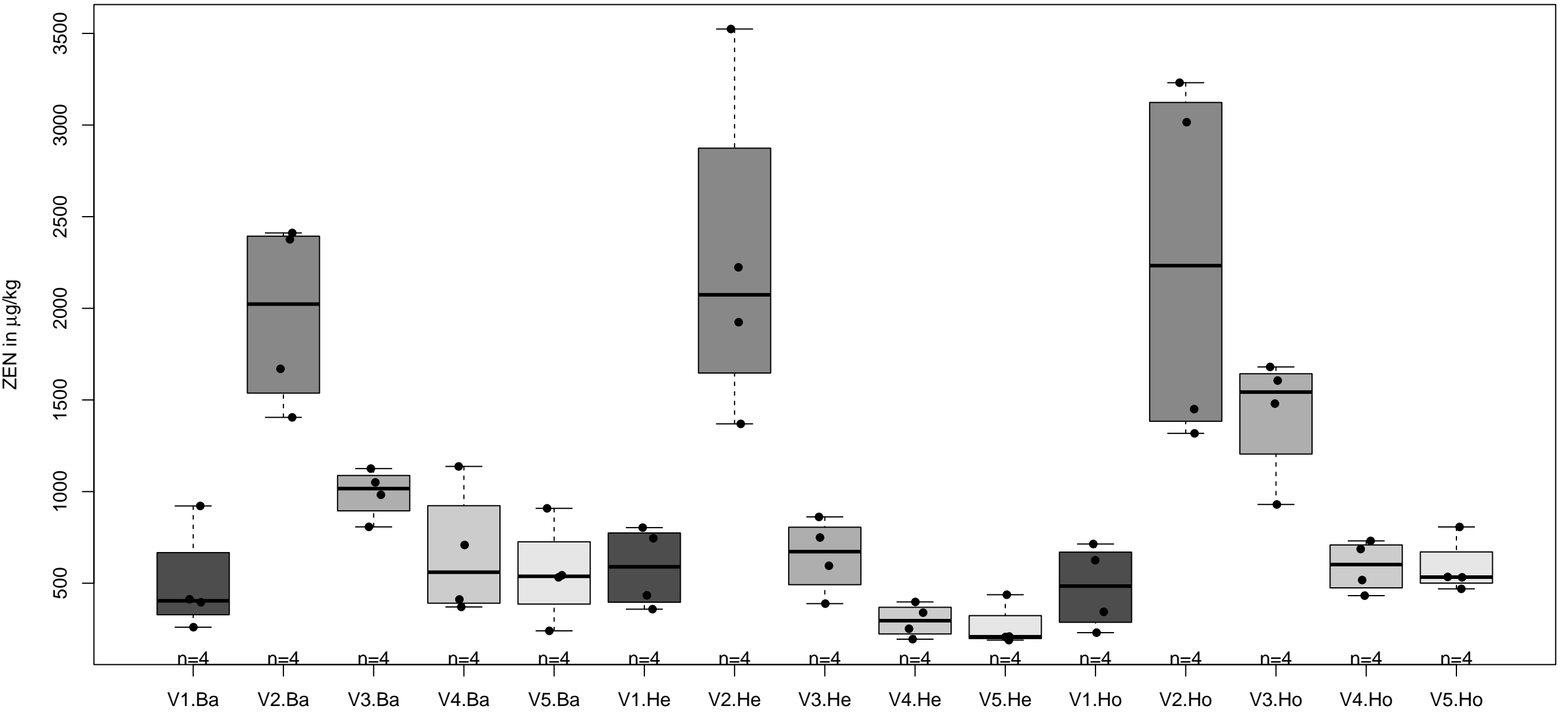

Supplement: Supplementary file 1 — Supporting Information [file BIMJ-67-e70019-s001.zip › Figures/bp_birr.pdf]

Number of micronuclei per 2000 cells

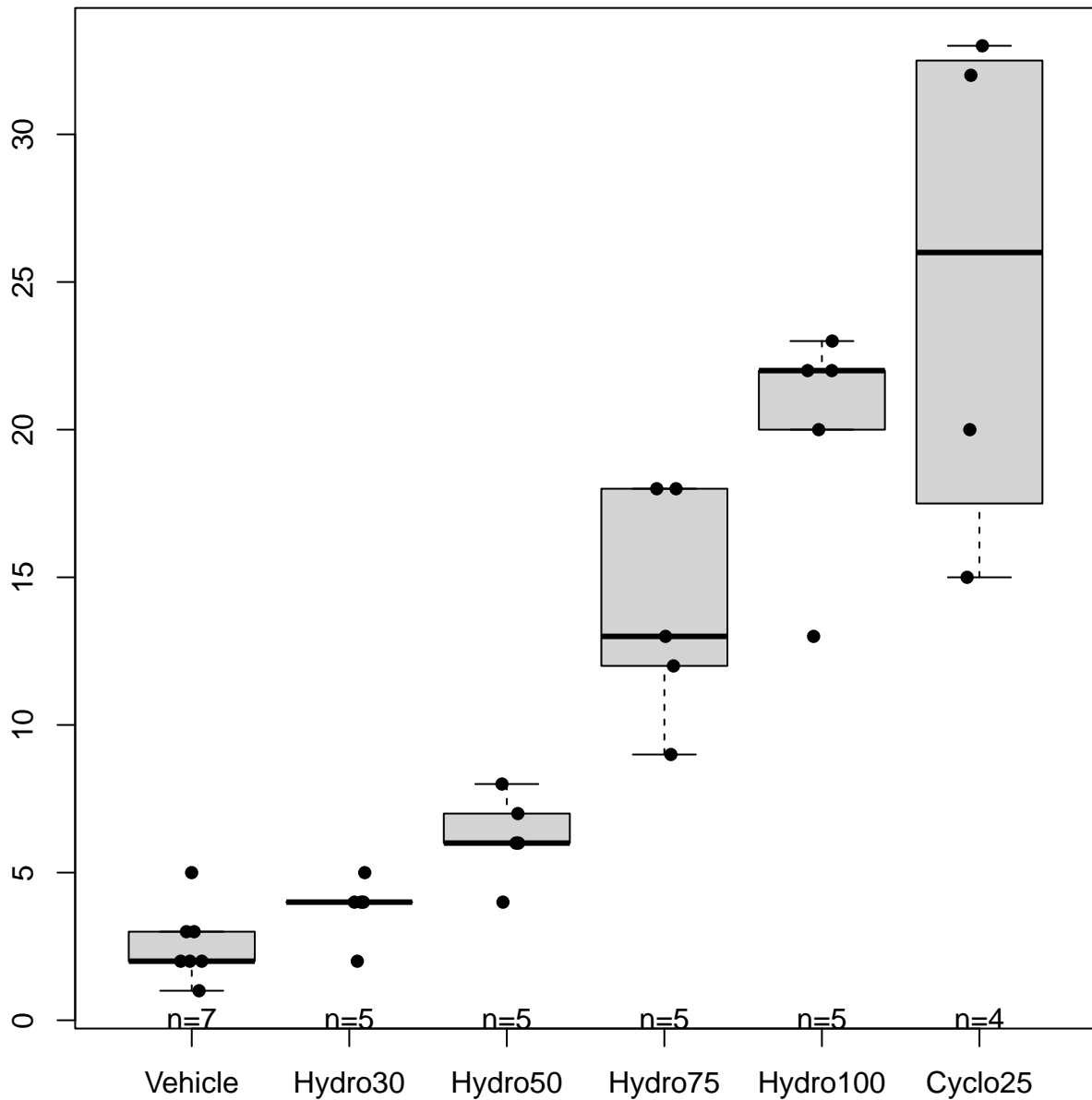

Supplement: Supplementary file 1 — Supporting Information [file BIMJ-67-e70019-s001.zip › Figures/bp_muta.pdf]

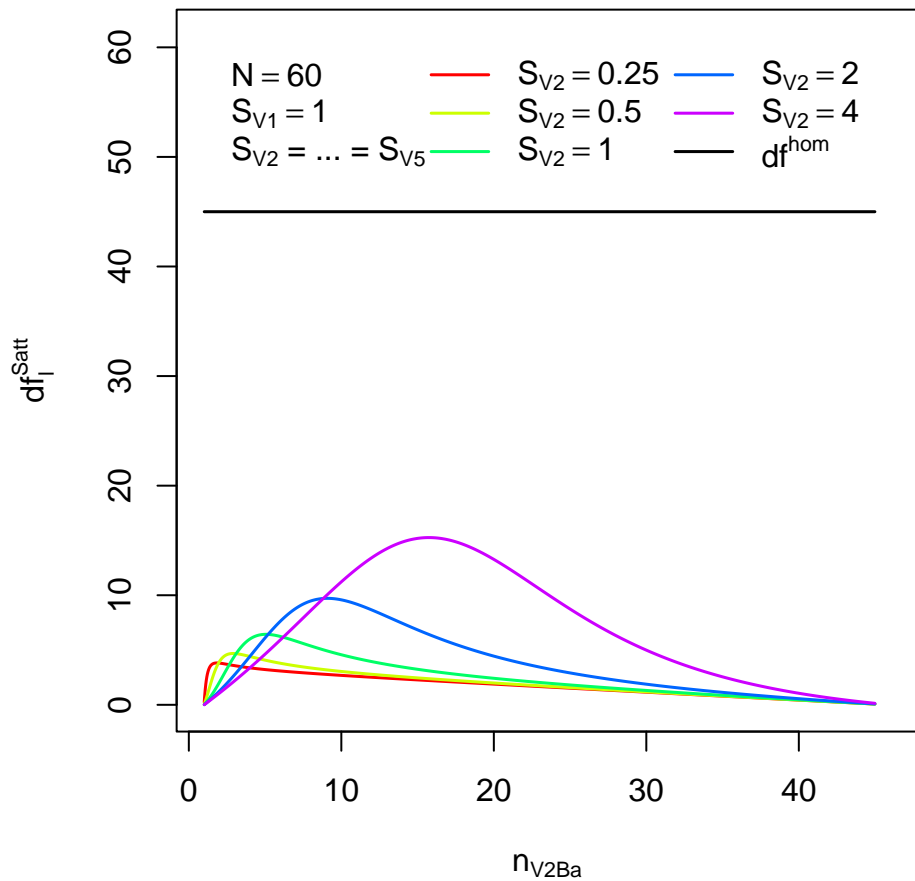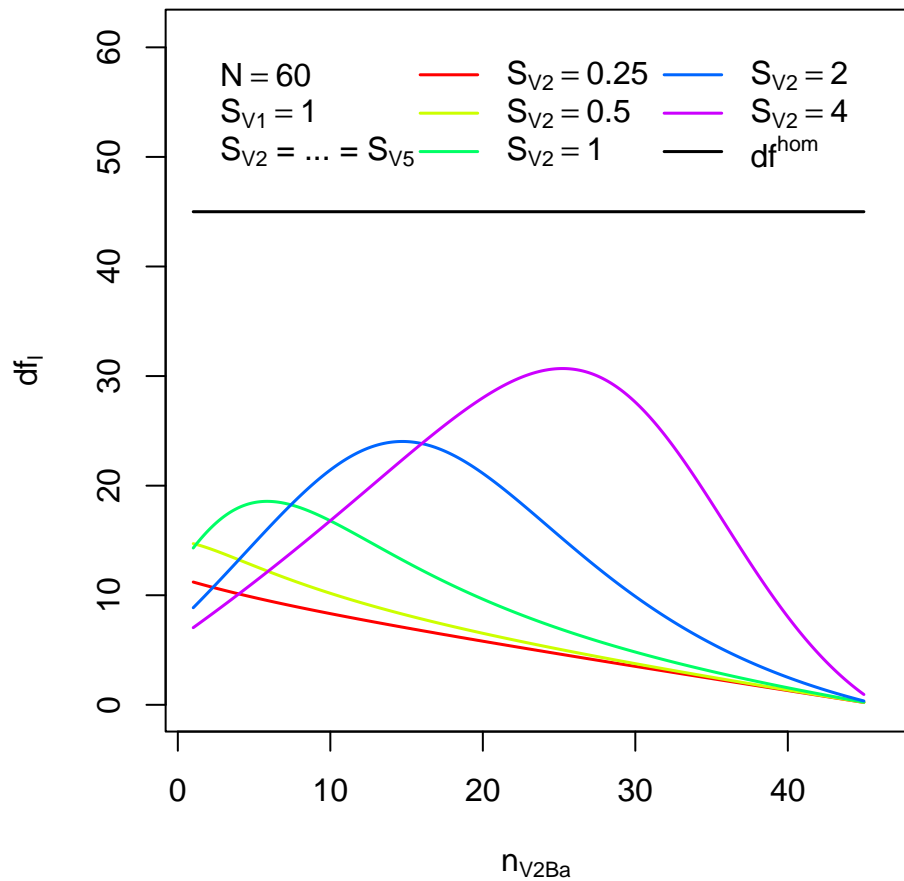

Supplement: Supplementary file 1 — Supporting Information [file BIMJ-67-e70019-s001.zip › Figures/df_PIaPH.pdf]

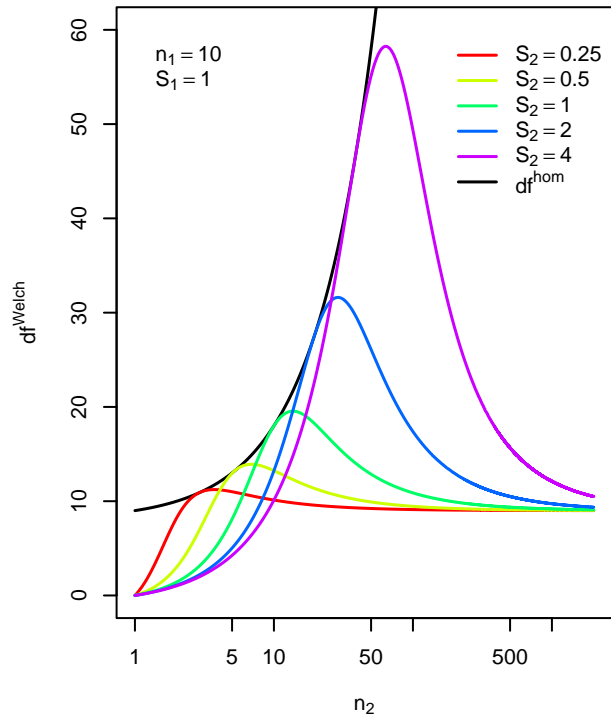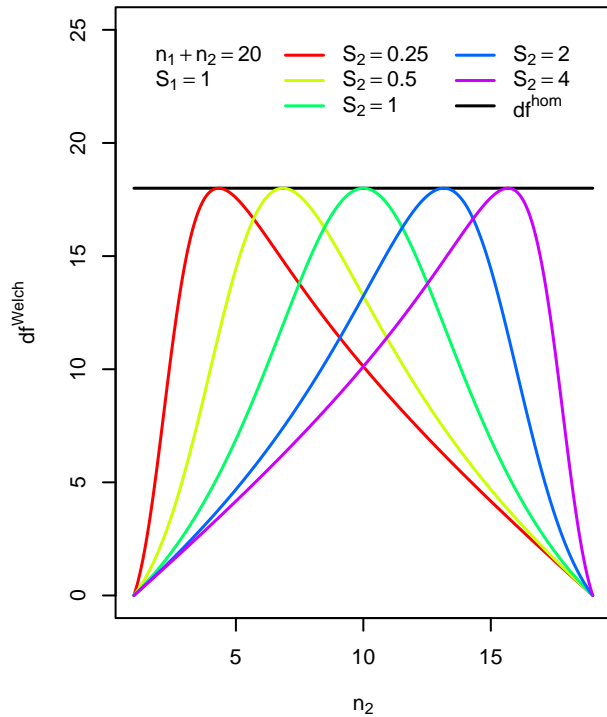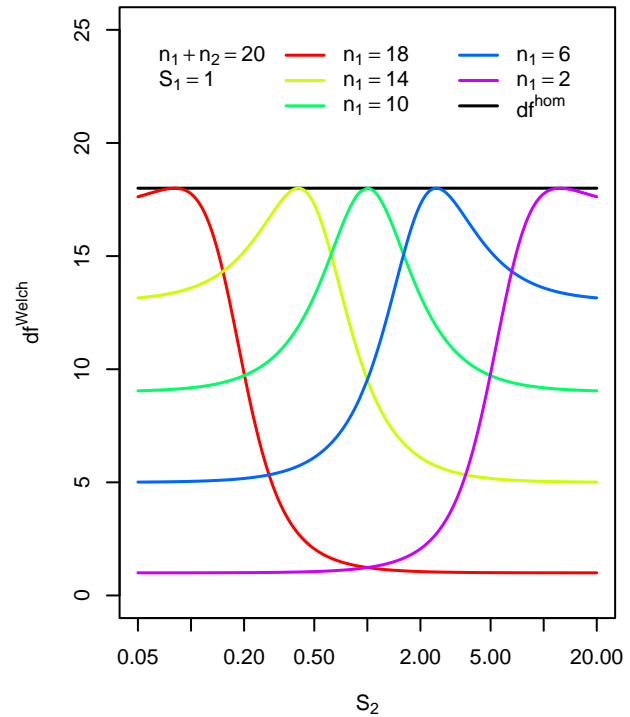

Supplement: Supplementary file 1 — Supporting Information [file BIMJ-67-e70019-s001.zip › Figures/df_Welch.pdf]

Setting a)

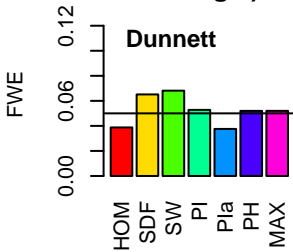

Setting b)

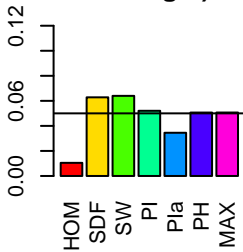

Setting c)

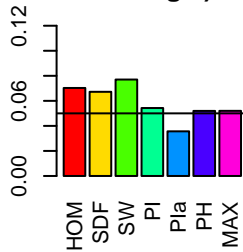

Setting d)

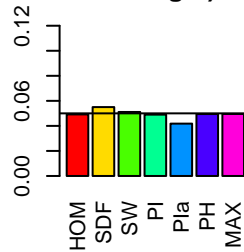

Tukey

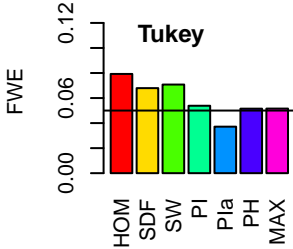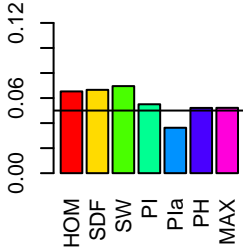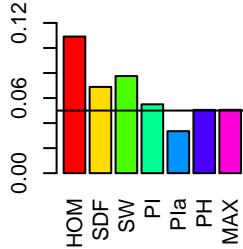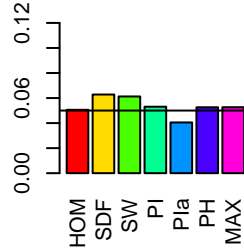

Williams

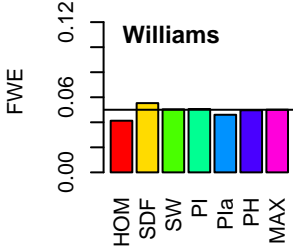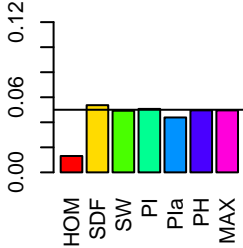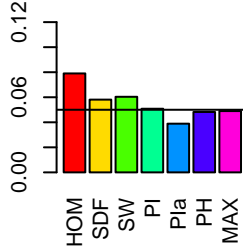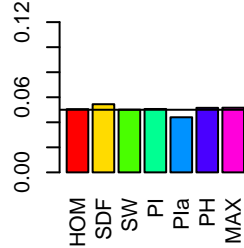

Average

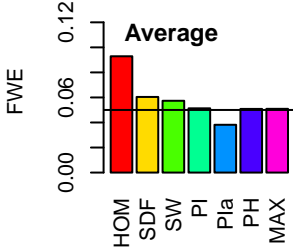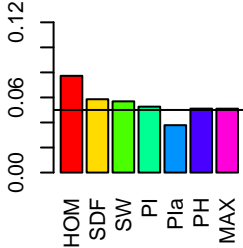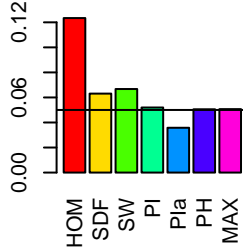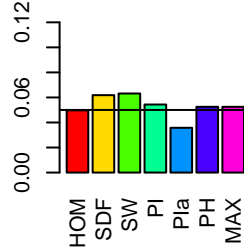

User-def.

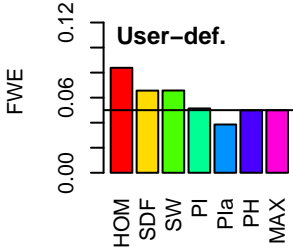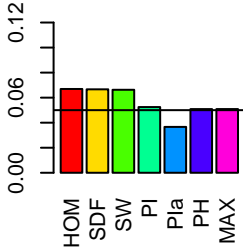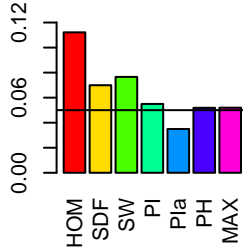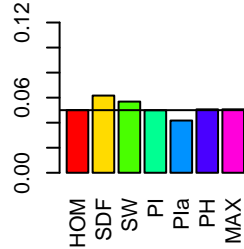

Supplement: Supplementary file 1 — Supporting Information [file BIMJ-67-e70019-s001.zip › Figures/fwe.pdf]

**Setting a)**

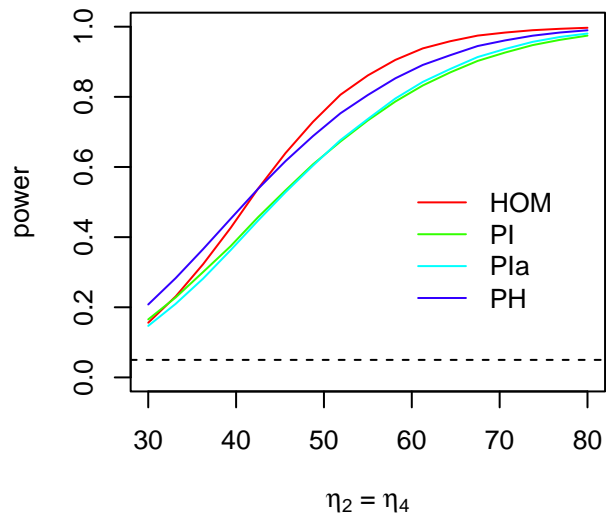

**Setting b)**

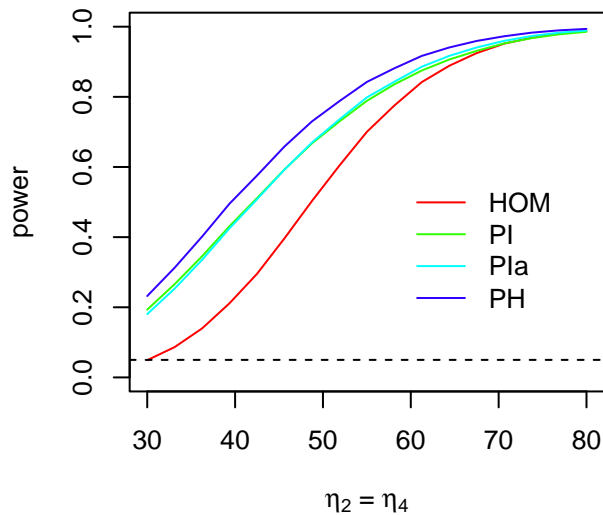

**Setting c)**

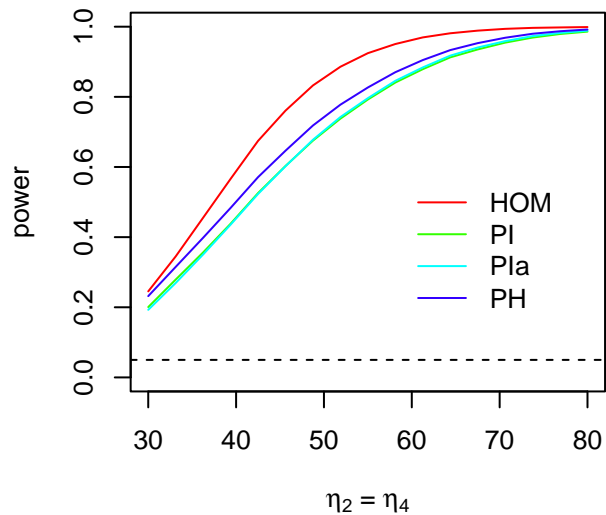

**Setting d)**

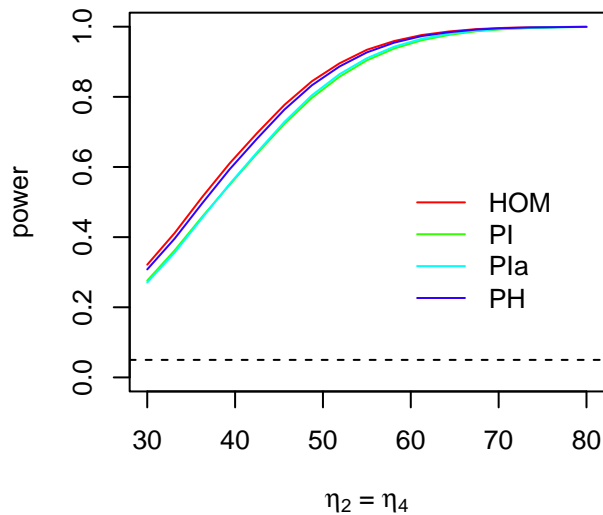

Supplement: Supplementary file 1 — Supporting Information [file BIMJ-67-e70019-s001.zip › Figures/parthetmctdiff_pow6Dunnett.pdf]

**Setting a)**

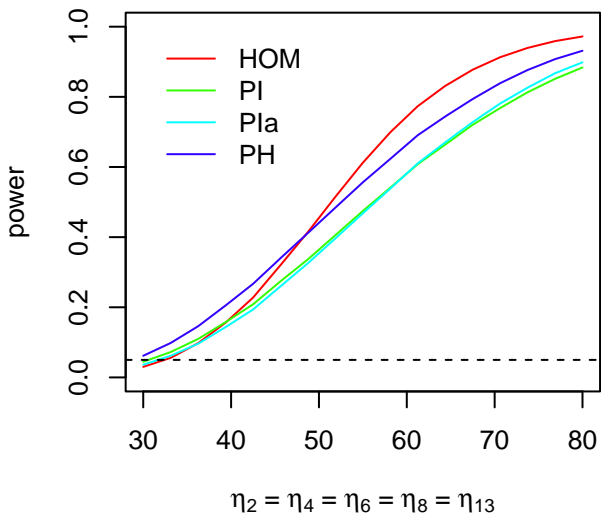

**Setting b)**

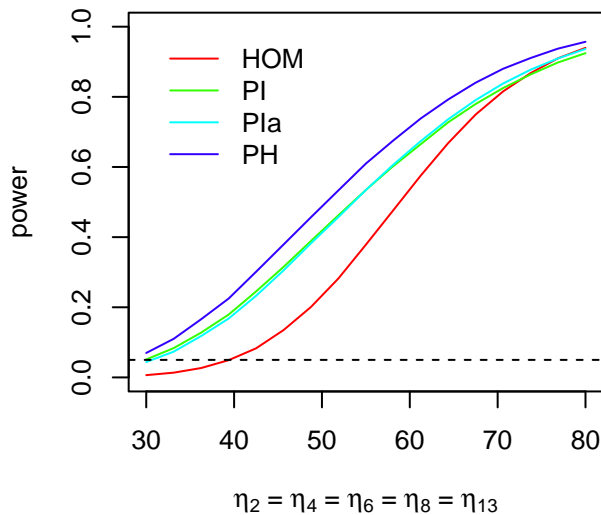

**Setting c)**

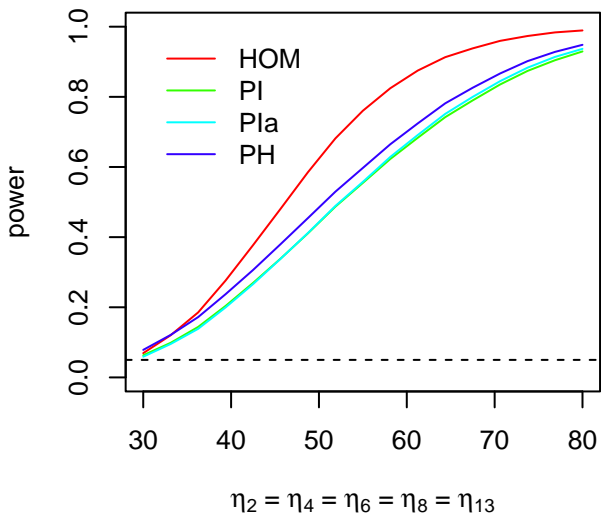

**Setting d)**

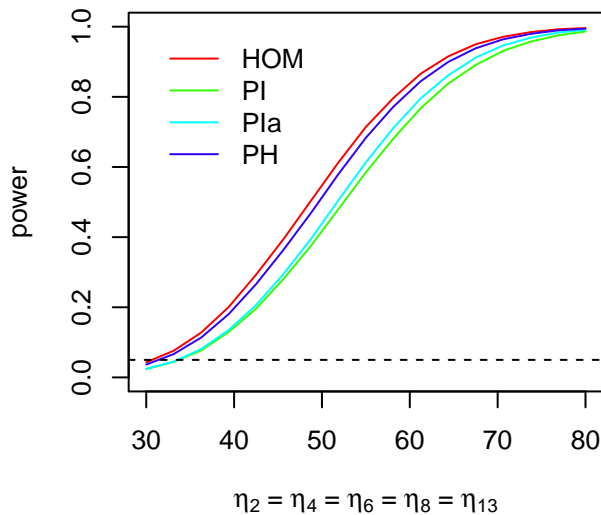

Supplement: Supplementary file 1 — Supporting Information [file BIMJ-67-e70019-s001.zip › Figures/parthetmctdiff_pow6Tukey.pdf]
